# Convergence Analysis of Cross-Province Human Well-Being in China: A Spatiotemporal Perspective

**DOI:** 10.3390/ijerph20031858

**Published:** 2023-01-19

**Authors:** Lei Jiang, Yuan Chen, Wenjie Liang, Bo Zhang

**Affiliations:** 1 School of Geography and Remote Sensing, Guangzhou University, Guangzhou 510006, China; 2 Guangdong Provincial Center for Urban and Migration Studies, Guangzhou 510006, China; 3 School of Economics, Jinan University, Guangzhou 510610, China; 4 Southern Marine Science and Engineering Guangdong Laboratory (Zhuhai), Zhuhai 519000, China

**Keywords:** human well-being, green economy, environmental sustainability, comprehensive evaluation method, spatial spillover, spatial econometrics, convergence analysis

## Abstract

China’s economy has been experiencing a new development mode that emphasizes an environmentally friendly green economy and high living standards. The concept of human well-being has become increasingly prominent in recent years to replace GDP per capita as an important indicator for evaluating happiness. In the context of the green economy, it is of great significance to incorporate environmental indicators for evaluating human well-being. To this end, this paper constructs a new human well-being evaluation indicator system including environmental sustainability, and then evaluates the well-being levels of 30 provinces in China from 2011 to 2020 using a comprehensive evaluation method. Then, various statistical methods and visualization methods are used to deeply analyze the spatiotemporal changes in the well-being scores of Chinese provinces during the sample period. Finally, the spatial convergence model was used to verify if cross-province well-being scores would converge to a common steady state. The findings are as follows. (1) The scores of the environmental sustainability subsystem greatly vary from province to province. This is because the local governments have attached great importance to the construction of green ecological civilization in recent years, thus increasing the investment in protecting the ecological environment. (2) From temporal dimensions, overall human well-being scores of 30 provinces slightly increased year after year. In geography, eastern provinces have the highest human well-being scores, followed by northeast, northwest, and southwest provinces. (3) In terms of the scores of the four subsystems, we find that nearly all provinces have their advantages and disadvantages. (4) From the results of the spatial convergence models, both absolute and conditional β convergence have been verified, indicating that the human well-being of all provinces will converge to the common steady state in the future.

## 1. Introduction

Since the reform and opening up in 1978, China’s economic development has undergone dramatic growth. In 2010, China’s total GDP surpassed that of Japan for the first time, ranking second in the world. However, with the enrichment of people’s material life, it becomes clearer that the single pursuit of GDP is no longer China’s most important goal. This is because economic development measured as GDP is an evaluation tool with only one dimension, which cannot comprehensively reflect the demand of people’s pursuit for a better life. The concept of human well-being as a research topic has started to gain momentum among the public. Promoting human well-being has also been repeatedly highlighted by the Chinese government in its administrative services. In the recent “Report on the Work of the Chinese Government”, the effective ways to improve people’s human well-being include basic infrastructure construction in rural areas, sufficient employment opportunities for these low-income groups, offering high-quality education and public health service, increasing life expectancy, optimizing services for children and the aged, improving the credibility of society, and maintaining a safer social environment. These measures indicate that well-being not only refers to the current state of people but also underlines the importance of the sustainable development goals (SDGs) enacted by the United Nations.

The concept of human well-being (well-being hereafter) was first proposed in the 1950s by the international community in response to the single pursuit of GDP growth in many North American and West European countries after World War II [1]. GDP as an important indicator for socioeconomic development is insufficient to measure the life quality of individuals. Therefore, the primary research about well-being focuses on how the part that GDP may not be fully reflected. The recent development on the topic of well-being articulates that well-being refers to people’s satisfaction with their current lifestyle, state, and the pursuit of a higher life value [2].

In a more general sense, well-being can be both subjective and objective. Subjective well-being usually pertains to comprehensive evaluations of people’s perceptions of their lives such as the emotions, moods, and cognitive judgments of their physical and mental conditions [3,4]. Based on cultural psychology, Andrews and Withey [5] proposed three basic pillars of subjective well-being which included life satisfaction judgments, positive affect, and negative affect. These three pillars may sometimes be controversial. For instance, Diener et al. put forward that positive and negative effects are always closely correlated to each other spatiotemporally. The mechanism can be that one may observe an increase in positive effects while a decline in the negative ones occurs. Moreover, it has been well documented that demographic features such as age, gender, race, employment, education, religion, marriage, and family are all important determinants that influence subjective well-being [6]. Compared to subjective well-being, objective well-being refers to the material and social attributes that are likely to influence people’s holistic needs such as basic survival needs, and economic and environmental needs that can be measured objectively [7]. The definition of objective well-being is relatively difficult; therefore, objective well-being is mainly defined by its dimensions [8]. In other words, objective well-being would be better defined with criteria. In particular, based on research by the Organization for Economic Co-operation and Development (OECD), the United Nations Development Programme (UNDP), and the Italian Statistics Bureau (ISTAT), Voukelatou et al. [8] identified six dimensions, namely, socioeconomic conditions, holistic health, employment opportunities, environmental circumstances, security, and politics to represent objective well-being. The above-mentioned research indicates that the concept of well-being is multidimensionally constructed and the measurement of well-being is contextually determined and strongly influenced by how to define and measure it.

The multidimensional nature of well-being signifies that the level of well-being should be evaluated through multiple evaluation index systems. Subjective well-being advocates that the evaluation criteria of well-being are people’s inner feelings and situational experiences. In the subjective well-being evaluation index system, questionnaire surveys are mostly used to carry out personal and emotional well-being evaluation research. The main subjective well-being assessment methods include the Satisfaction with Life Scale (SWLS) [3,9] and the Pemberton Happiness Index [10]. The index system methods for objectively evaluating well-being mainly include the Sustainable Economic Wellbeing Index [11], the Real Savings Method [12], the Material Quality of Life Index [13], and the Real Progress Index [14]. The Human Development Index (HDI) is one of the most widely used index systems proposed by the United Nations Development Programme in 1990 Development Index, which includes three indicators, namely, income, education, and life expectancy. It can be summarized that the objective welfare evaluation system focuses on how social development provides people with better services, materials, and a higher quality of life. Although the focuses of subjective and objective well-being are different, it has been argued that there is a strong correlation between the two [15,16]. Besides, the evaluations of subjective and objective well-being are not antagonistic relationships but complementary to each other, therefore, well-being levels should be evaluated in the combination of the two [17]. For example, Costanza et al. [18] constructed a mixed index system for evaluating the quality of life and well-being from a multi-scale and multi-dimensional perspective. In addition, the Kingdom of Bhutan has adopted gross national happiness, a measure of both subjective and objective measures of well-being, to replace GDP as a measure of a country’s development [19].

The Millennium Ecosystem Assessment (2003, 3) project proposed the classic definition of well-being which “has multiple constituents, including basic material for a good life, freedom and choice, health, good social relations, and security” and “the constituents of well-being, as experienced and perceived by people, are situation-dependent, reflecting local geography, culture, and ecological circumstance”. This definition shows the close relationship between the ecological environment and human well-being which constitute the core agenda of sustainable science. A more recent definition given by the OECD [20] emphasizes not only its multi-dimensional nature but also indicates the interactions of multiple components of well-being. For instance, well-being reflects the human-nature relations and how humans utilize the diverse basic substances in the ecosystem for better life experiences, more importantly, how humans could sustain their ability to maintain their health conditions, social support, freedom of choice, and safety [21] in a broader sense. It is a human experience that includes the process of developing and utilizing various basic substances in the natural ecosystem for a better life, and the freedom and ability to make choices about physical health, social relations, cultural identity, belonging, security, and self-realization. This means that the connotation of well-being contains the interactions between humans and the surrounding environment.

It is well established that the relationships between well-being and the ecosystem and environmental service stand for the interactions between nature and society [22,23]. In general, ecosystems can promote people’s physical and mental health and make people happier, therefore, contributing to people’s holistic well-being [24,25,26]). The benefits of an ecosystem service to human well-being are various while the core of it is human–nature relationships. The benefits of ecosystem services to human well-being are summarized in the Millennium Ecosystem Assessment [27] as provisioning, regulating, supporting, and cultural services which cover a wide range of the important products that humans can obtain from their environmental surroundings. For instance, Pedersen et al. [28] find that residents’ perception of their quality of life has a strong impact on the values of cultural ecosystem service. Yee et al. [29] put forward that ecosystem services can not only provide benefits to human well-being but also are conducive to community well-being through a series of elements such as cultural fulfillment and connection to nature. Aguado et al. [30] demonstrate how sociocultural perceptions of human–nature relationships are significant to sustainable ecosystem management. It is important to notice that the achievement of a sustainable ecosystem is the key to human well-being which contains both perception and objective indicators.

In summary, although researchers have realized that the ecosystem and the environment have become important factors affecting human well-being, enough attention to incorporating ecological environment-related indicators into the human well-being evaluation system has not yet been received. Therefore, one aim of this paper is to improve the human well-being evaluation system by expanding the ecosystem and environment-related indicators based on the existing literature. Moreover, it has been recognized that there are huge disparities in human well-being levels across province in China. Hence, a question arises if they can converge to a common steady state in the future. Although some researchers have investigated convergence of well-being across nations or regions (see, amongst others, [31,32,33]), to the best of our knowledge, they have neglected spatial spillovers. Technically, observations are not evenly distributed in space, but usually present spatial clustering, which should be considered when verifying the convergence of well-being across regions. Otherwise, biased conclusions may result. Hence, to answer this question, we build a comprehensive evaluation model including four subsystems, namely, basic needs, social welfare, culture and education, and environmental sustainability, and 36 specific indicators to comprehensively and objectively evaluate the well-being scores of 30 provinces in China from 2011 to 2020, and then apply spatial convergence models to verify if the well-being scores of these provinces converge to a common state.

The contributions of this study are threefold. First, considering data availability, we expand evaluation indicators and adopt an entropy weight method to calculate the well-being scores of 30 provinces from 2011 to 2020. After the calculation, various statistical graphs and mapping methods are used to analyze the spatiotemporal distribution and variations of the four subsystem scores and the total well-being scores of 30 provinces, including run charts, stacked column charts, parallel coordinate plots, maps, and kernel density charts. Third, there are significant spatial differences in well-being across the province but well-being scores in proximate provinces tend to be clustered, namely, spatial clustering. In other words, spatial spillover works and plays an important role in the well-being levels of both the local and neighboring provinces. Otherwise, the omission of spatial spillover may lead to biased conclusions and even undermine the foundation of the convergence analysis in our study. In this sense, this study introduces the concept of convergence derived from economics and incorporates spatial spillover effects to build spatial convergence models to verify if the low well-being provinces will catch up with those with high scores. Technically, the most important aim is to test if the well-being scores of 30 provinces can converge to a common steady state in the future. Finally, policy recommendations based on the main results are proposed. The findings of this paper are important for balancing the differences in well-being across provinces in China.

## 2. Models and Data Sources

### 2.1. Comprehensive Evaluation Method

Based on data availability, in this study, we refer to the relevant studies (e.g., [34,35]) and construct the well-being evaluation indicator system, which includes four subsystems and 36 indicators, namely, basic needs (BN), social welfare (SW), culture and education (CE), and environmental sustainability (ES). They are listed in Table 1.

The basic needs subsystem mainly contains economic indicators and basic physical supply indicators. Among them, GDP per capita and consumption of residents are widely regarded as the important socioeconomic factors that affect human well-being. In addition, basic physical supplies such as water resources per capita, food production, and meat production are the basic conditions to meet the demand for survival and life are objective indicators to assess well-being.

In the social welfare subsystem, the number of traffic accident deaths per 10,000 people is a reflection of traffic safety. In addition, indicators such as the number of people per 10,000 people receiving work injury benefits, the percentage of working employees participating in pension insurance, and the number of beds in medical and health institutions per 10,000 people describe the social welfare for urban and rural residents, which are important social welfare factors affecting well-being.

The culture and education subsystem includes a wide range of relevant indicators, such as the number of museum institutions and the number of general higher education schools per 10,000 people. As the importance of education and knowledge increases, educational resources should also be included in the human well-being indicator system.

In the environmental sustainability subsystem, it contains environmental investment, environmental pollution, and environmental management indicators. Specifically, environmental investment considers the completed investment in ecological construction and protection per capita. Environmental pollution indicators mainly refer to pollutants, such as wastewater discharge, sulfur dioxide, and carbon oxide emissions. Environmental management indicators incorporate the total area of planted forests and the number of nature reserves. Environmental quality has increasingly become an important factor affecting well-being. This is because better environmental quality draws much more attention when income increases.

### 2.2. Standardization of Indicators

From Table 1, we notice that these heterogeneous indicators have different units and cannot be merged into a unified evaluation system. In addition, they can also be divided into two types, namely, positive (i.e., the higher the better) and negative (the lower the better) indicators. Hence, to eliminate the differences in units and orders of magnitude between the different types of indicators and make heterogeneous indicators homogenous, we adopt a standardization method. It is shown as follows [36].

(1) $$\mathrm{Positive}\;\mathrm{indicators}\text{:}\;x_{\mathrm{ij}}^{\mathrm{Po}}=(x_{\mathrm{ij}}-\mathrm{Min}\left(x_{\mathrm{ij}}\right))/\left(\mathrm{Max}\left(x_{\mathrm{ij}}\right)-\mathrm{Min}\left(x_{\mathrm{ij}}\right)\right)$$

(2) $$\mathrm{Negative}\;\mathrm{indicators}\text{:}\;x_{\mathrm{ij}}^{\mathrm{Ne}}=(\max\left(x_{\mathrm{ij}}\right)-x_{\mathrm{ij}})/\left(\mathrm{Max}\left(x_{\mathrm{ij}}\right)-\mathrm{Min}\left(x_{\mathrm{ij}}\right)\right)\;$$

where Min and Max represent the minimum and maximum values of the indicator j, respectively. $x_{\mathrm{ij}}$ denotes the jth indicator of province i while $x_{\mathrm{ij}}^{\mathrm{Po}}$ and $x_{\mathrm{ij}}^{\mathrm{Ne}}$ are the standardized positive and negative indicators j of province i, respectively. For simplicity, both of them can be labeled $x_{\mathrm{ij}}^{\prime }$.

### 2.3. Entropy Weight Method

The entropy weight method, derived from information entropy first proposed by Shannon in 1948, has been widely applied in empirical studies. The larger the entropy value, the more complex the system is, and vice versa. The entropy weight method is introduced as follows [36].

(3) $$P_{\mathrm{ij}}=\frac{x_{\mathrm{ij}}^{\prime }}{\Sigma_{i=1}^{n}x_{\mathrm{ij}}^{\prime }}$$

(4) $$e_{j}=-\frac{1}{\mathrm{logn}}\Sigma_{i=1}^{n}P_{\mathrm{ij}}\times {\mathrm{logP}}_{\mathrm{ij}}$$

(5) $$w_{j}=\frac{\left(1-e_{j}\right)}{\Sigma_{j=1}^{n}\left(1-e_{j}\right)}$$

where log denotes logarithm transformation. n is the number of provinces. $w_{j}$ represents the weight of indicator j.

After obtaining the weights using the entropy method, it is easy to calculate the scores of four subsystems (i.e., basic needs, social welfare, culture and education, and environmental sustainability) and the well-being scores of 30 provinces from 2011–2020 by the summation of the corresponding weights multiplied with the standardized values. The formula is expressed as follows.

(6) $$U_{m}=\Sigma_{j=1}^{n}w_{j}x_{\mathrm{ij}}^{\prime }$$

where the subscript m (m = 1, 2, 3, and 4) denotes the four subsystems, namely, basic needs, social welfare, culture and education, and environmental sustainability.

Similarly, we repeat the same procedure to calculate the well-being scores of 30 provinces from 2011 to 2020 based on the four subsystems.

### 2.4. Global Autocorrelation Analysis: Moran’s I

Traditional statistical methods assume that observations are independent of each other. However, geo-referenced data usually violate the assumption since the spatial distribution of the observations is not random. For our study, we can observe that the well-being scores of 30 provinces are not randomly distributed in space. Hence, in this research we employ a widely used spatial autocorrelation approach, namely, Moran’s *I* first proposed by Moran [37], to verify if there is spatial autocorrelation of the well-being scores of 30 provinces. It is expressed below.

(7) $$I=\frac{n}{\Sigma_{i=1}^{n}\Sigma_{j=1}^{n}W_{\mathrm{ij}}}\frac{\Sigma_{i=1}^{n}\Sigma_{j=1}^{n}W_{\mathrm{ij}}\left(y_{i}-\bar{y}\right)\left(y_{j}-\bar{y}\right)}{\Sigma_{i=1}^{n}{\left(y_{i}-\bar{y}\right)}^{2}}$$

where *I* is Moran’s *I*; y_i_ denotes the well-being score of province i; and $\bar{y}$ is the average value. n represents the number of Chinese provinces, that is, 30 in this study. An n × n spatial weights matrix W in the equation is used to describe the spatial arrangement of these provinces. In other words, it can capture spatial interaction effects among the provinces. In this study, we consider the two most common spatial weights matrices, namely, rook contiguity (Rook hereafter) and k-nearest neighbor matrices. The elements of Rook are equal to 1 if two provinces have common borders and 0 otherwise. In addition, k denotes the number of nearest neighbors. In this study, k is assumed as 4 (K4 hereafter).

The range of Moran’s *I* value is restricted to the interval [−1, 1]. If it is significant and positive, it indicates spatial clustering. Moreover, if it is significant and negative, it implies spatial dispersion. Moreover, if Moran’s *I* value is statistically insignificant, it suggests a random distribution.

### 2.5. Kernel Density Estimation

Convergence is generally divided into *σ* convergence and β convergence. *σ* convergence is defined as the decrease in the dispersion across 30 provinces in this study. In most empirical studies, the standard deviation or the coefficient of variation (CV) has been widely used (e.g., [31,32]). However, they also suffer from some shortcomings. The kernel density estimation as a more adequate approach has gained more popularity to measure *σ* convergence in recent years since it has two main advantages. One is to allow us to analyze the distribution as a whole and the second is a detailed forecast of the long-run distribution. Kernel density estimation was first proposed by Rosenblatt [38] and Paren [39]. It is expressed below.

(8) $$f\left(x\right)=\frac{1}{\mathrm{nh}}\Sigma_{i=1}^{n}K\left(\frac{x-X_{i}}{h}\right)$$

where f(x) denotes density function. n is the number of provinces. K refers to the kernel weighting function that integrates to 1. Last, h is the bandwidth. In this study, we adopted Epanechinikov kernel [40] and Silverman’s bandwidth [41] to estimate the kernel densities of the well-being scores of 30 provinces from 2011 to 2020.

### 2.6. Spatial β Convergence Model

The basic concept of β convergence was originally used for the convergence test of per capita income. It assumes that rich economies grow slower than poor economies in terms of GDP per capita growth rate [42,43,44]. This study borrows the idea of β convergence to test whether provinces with low well-being scores can catch up with provinces with high well-being scores and will eventually converge to the common steady level.

β convergence is divided into two types, namely, absolute and conditional β convergence. Unlike the non-parametric *σ* convergence, β convergence is verified using a parametric estimation approach. Specifically, a regression model is used to test the negative relationship between the initial level of well-being score and its growth rate. If the estimated coefficient β is significant and negative, it indicates that there is β convergence. The absolute β convergence model is introduced as follows.

(9) $${\mathrm{logWBG}}_{\mathrm{it}}=\alpha +{\beta \mathrm{logWB}}_{\mathrm{it}-1}+\varepsilon_{\mathrm{it}}$$

where ${\mathrm{logWBG}}_{\mathrm{it}}$ is the growth in the well-being score of province i at year t in logs. ${\beta \mathrm{logWB}}_{\mathrm{it}-1}$ denotes the lagged well-being score in logs. α is the constant term and ε is the error term.

Different from absolute β convergence, conditional β convergence allows different subsets of provinces to converge to different steady states, conditional on province-specific characteristics. Hence, an easy way to build the conditional β convergence model is to control for cross-sectional fixed effects based on the absolute β convergence model. Hence, it can be written as below.

(10) $${\mathrm{logWBG}}_{\mathrm{it}}=\alpha +{\beta \mathrm{logWB}}_{\mathrm{it}-1}+\mu_{i}+\gamma_{t}+\varepsilon_{\mathrm{it}}$$

where $\mu_{i}$ denotes the province-fixed effects that are used to control for province-specific and time-invariant variables in the convergence model. Similarly, we also need to control for time-fixed effects $\gamma_{t}$ to avoid possible omitted-variables bias. Hence, it is usually called the two-way fixed effects conditional β convergence model.

As mentioned earlier, we find that the well-being scores of proximate provinces tend to have similar values, indicating spatial spillover or spatial clustering. The results of Moran’s *I* test also confirm significant spatial autocorrelation. Since it is ignored in the traditional conditional β convergence model, biased estimates may be obtained even if it may undermine the foundation of the convergence analysis. To overcome this drawback, we incorporate spatial spillover effects and then build the two-way fixed effects spatial conditional β convergence model [45]. It is written as follows.

(11) $${\mathrm{logWBG}}_{\mathrm{it}}=\alpha +{\rho \mathrm{WlogWBG}}_{\mathrm{it}}+{\beta \mathrm{logWB}}_{\mathrm{it}-1}+\mu_{i}+\gamma_{t}+\varepsilon_{\mathrm{it}}$$

where ${\mathrm{WlogWBG}}_{\mathrm{it}}$ is the spatially lagged dependent variable that is used to capture the spatial spillovers. W is also a spatial weights matrix, similar to that in Moran’s *I* test. In addition, ρ, called the spatial autoregressive coefficient, is an unknown parameter to be estimated. All other variables are the same as in Equation (10).

### 2.7. Data Sources

The data for the comprehensive evaluation of the well-being scores of 30 Chinese provinces from 2011 to 2020 were obtained from the China Statistical Yearbooks. It should be noted that due to data unavailability, some data for Tibet are missing.

## 3. Spatiotemporal Variations of Well-Being Scores

### 3.1. Trends of Scores of Four Subsystems and Well-Being

**Figure 1:**
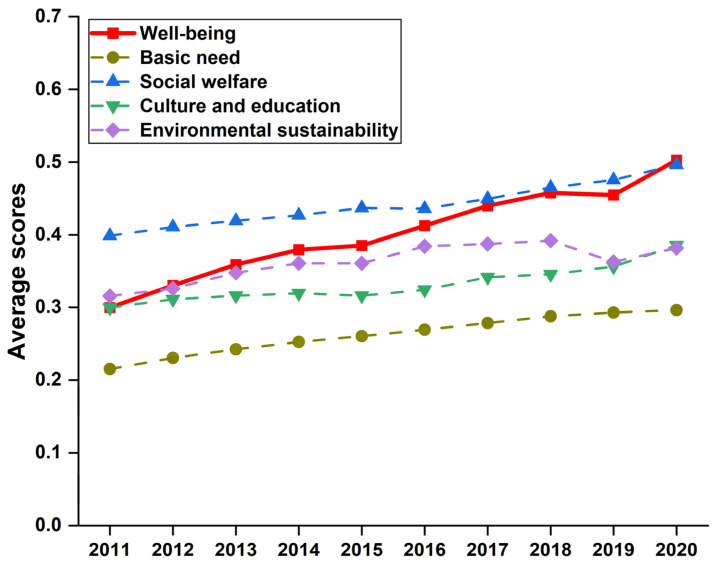
Trend of scores of four subsystems and well-being from 2011 to 2020.

The comprehensive evaluation method was utilized to calculate the well-being scores of 30 provinces from 2011 to 2020. We first present the trends of the average scores of four subsystems and well-being in Figure 1.

We observe that overall average values of the four subsystems and well-being during the sample period increase year by year, indicating that China’s human well-being has gradually improved over time. In other words, it also implies that China is experiencing the transition from rapid economic growth to people-oriented high-quality development.

Regarding the four subsystems, the social welfare subsystem has the highest average values, followed by the environmental sustainability, culture and education, and basic needs subsystems. It implies that the Chinese government has strengthened social welfare works to increase social welfare levels of all provinces in the recent decade. It should be noted that the environmental sustainability subsystem presents an increasing trend, but with an interruption in 2019. The main reason is that environmental investment in some provinces sharply declined caused by the COVID−19 pandemic.

Although the subsystems of culture and education and basic needs have lower values, they still witness increasing growth. On the one hand, China should pay much more attention to culture construction and education investment for the purpose of well-being improvements. On the other hand, the shortage of food supply may pose a threat to the well-being of most provinces. In other words, China should widen food supply channels through various measures, for example, the wide introduction of agricultural technologies and food imports from other countries to guarantee the food safety of China.

### 3.2. Scores of Four Subsystems across Provinces

**Figure 2:**
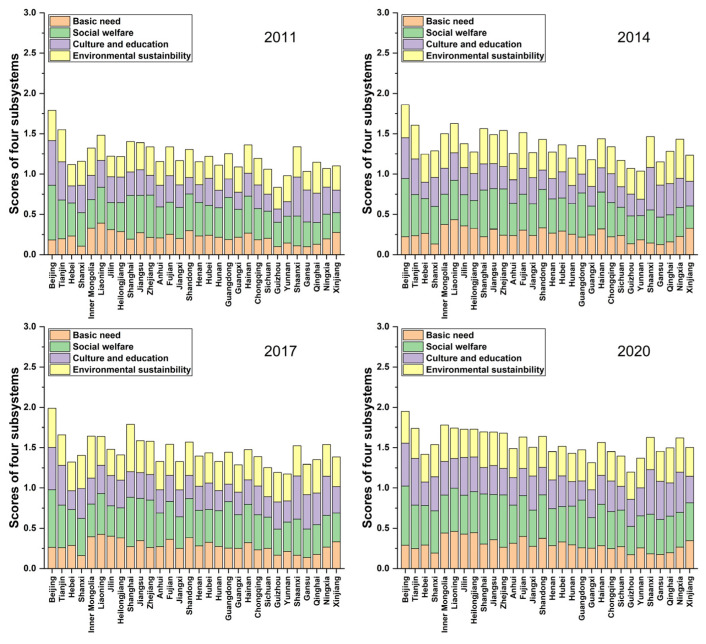
Scores of four subsystems in 2011, 2014, 2017, and 2020.

Next, we focus on the discussion about the score of each subsystem. It is presented in Figure 2 using a stacked column chart, where it comprises four columns stacked vertically, namely, the scores of four subsystems, one on another. The summation of four columns generally measures the well-being of each Chinese province. It should be noted that the theoretical maximum of the summation of the scores of the four subsystems is 4.

In 2011, we observe that Beijing has the highest column, indicating the highest well-being score. Among the subsystems, it can be found that the basic needs subsystem has a low value mainly because food products from agriculture are small in Beijing. Hence, it has long been dependent on food imports. In contrast, it has a high score in the culture and education subsystem, indicating advantages in the number of museums and universities. Shanghai, the second most important mega-city in China, has a similar structure. On the other hand, we find that Guizhou has the lowest scores of the four subsystems, indicating the lowest well-being score.

In 2014, it has a similar pattern to that in 2011. Still, the highest well-being province was Beijing while the lowest was not Guizhou, but Yunnan. It is mainly attributed to the culture and education subsystem improvement in Guizhou. In 2017, Shanghai performed the best since it had improved the four subsystems of well-being, notably environmental sustainability. This is because Shanghai has attached great importance to addressing the problems of industrial pollutants as environmental awareness increases over time. We also find that Shanghai is the second-best well-being region, just behind Beijing. In 2020, we witnessed that the four subsystems of all provinces had greatly improved, notably the western provinces, indicating that the national well-being level significantly increased.

**Figure 3:**
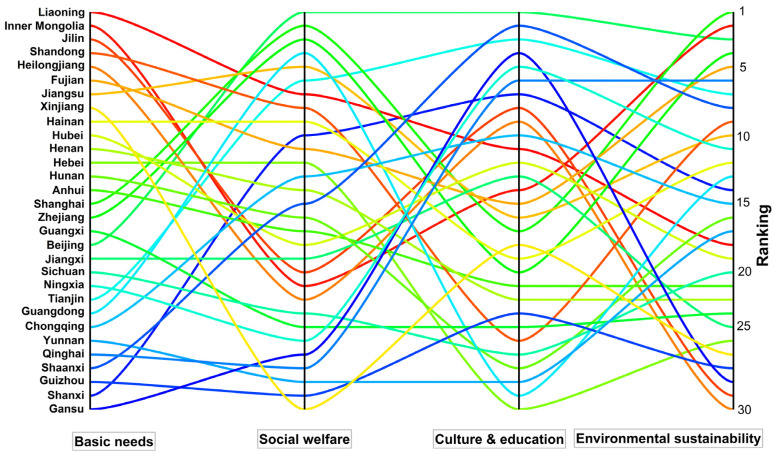
Rankings of subsystem scores of 30 provinces in 2020.

To better show the scores of the four subsystems of each province, a parallel chart was applied to display the advantages and disadvantages of each province when evaluating well-being (Figure 3). Specifically, we re-arranged the scores of each subsystem of 30 provinces into ascending order. The higher the ranking is, the better the subsystem level.

Figure 3 has four columns, which denote the rankings of 30 provinces in terms of the scores of the four subsystems. From column 1, we find that Liaoning ranks first in the basic needs subsystems, which is mainly attributed to sufficient food supply and a relatively high income. However, it has lower rankings in the other three subsystems. Inner Mongolia, Jilin, and Heilongjiang also outperformed the other provinces in the basic needs scores, but have low social welfare scores (column 2). Shanghai and Zhejiang had disadvantages in basic needs but outperformed others in terms of social welfare. A similar finding can also be applied to Tianjin, Guangdong, Chongqing, Shaanxi, and Shanxi. From column 3, we notice that the western Shaanxi, Shanxi, and Gansu provinces have relatively high rankings in terms of culture and education, which is mainly attributed to a large number of museums and universities. From column 4, we find that Yunnan has a relatively high ranking in terms of the environmental sustainability systems, which is mainly attributed to the better natural environment and less industrialization but has disadvantages in the other three subsystems. As a result, it has a low well-being score.

From Figure 3, we conclude that most provinces have both advantages and disadvantages. Provinces with high well-being levels usually at least have a disadvantage while low well-being provinces may suffer from several disadvantages. Hence, to improve well-being, much attention should be paid to all indicators, notably the low-score indicators. Otherwise, the disadvantages may be exacerbated and it is unlikely that the well-being scores of all provinces will converge to a common steady state. From the above analysis, it follows that the central government should play a key role in promoting trans-regional cooperation to eliminate the disadvantages and achieve the goal of a high well-being society.

### 3.3. Changes in Rankings of The Well-Being Scores of Provinces

**Figure 4:**
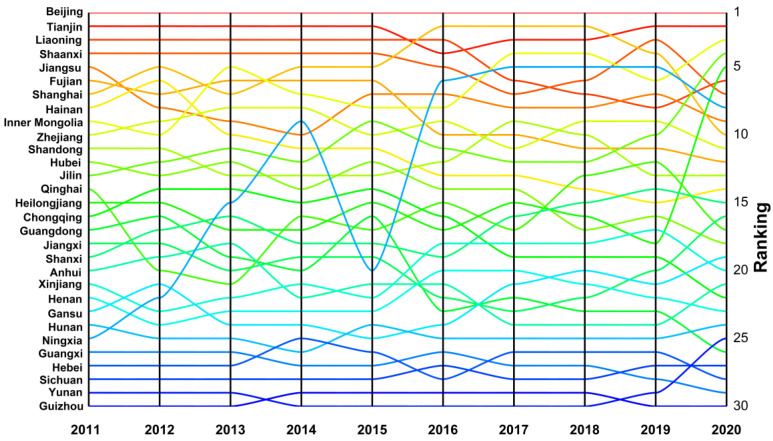
Changes in rankings of well-being scores of 30 provinces.

We also applied the entropy weight method to calculate the well-being scores of 30 provinces based on the scores of the four subsystems and then re-arranged the scores of 30 provinces into ascending order. Hence, the changes in rankings of the well-being scores of 30 provinces from 2011 to 2020 can be presented in Figure 4.

We observe that the rankings of most provinces had not changed much from 2011 to 2020. In other words, they slightly fluctuated during the sample period. Specifically, Beijing always tops the list since it has the highest well-being score, followed by Tianjin and Liaoning, Shaanxi. However, the three provinces declined from 2015 to 2020. The eastern provinces, such as Jiangsu, Fujian, Shanghai, Zhejiang, and Shandong usually outperformed because they had higher income, better social welfare, stronger culture and education, and more environmental investment. In contrast, for most of the western provinces, such as Gansu, Guangxi, Sichuan, Yunnan, and Guizhou, low well-being scores left them at the bottom of the ranking. It should be noted that Ningxia has significantly improved its ranking over time, which is primarily attributed to improvements in the social welfare system and increases in investments in culture and education.

### 3.4. Spatiotemporal Variations of Well-Being Scores

**Figure 5:**
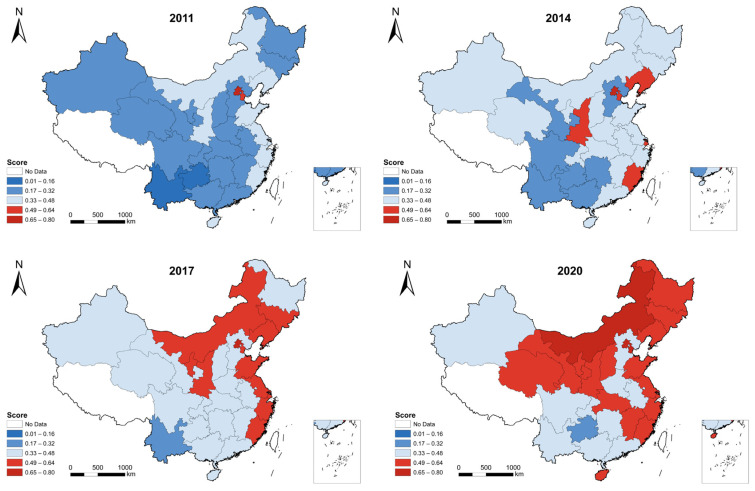
Spatial distribution of well-being scores in 2011, 2014, 2017, and 2020.

We adopted a geo-visualization technique to map the spatial distribution of the well-being scores of 30 provinces in 2011, 2014, 2017, and 2020. The distribution is shown in Figure 5.

In 2011, Beijing had the highest score because of outstanding social welfare and culture and education scores, indicating the highest well-being region, followed by eight eastern provinces, namely, Tianjin, Liaoning, Shandong, Jiangsu, Shanghai, Zhejiang, Fujian, and Hainan, and two western provinces, namely, Inner Mongolia and Shaanxi. The rest of the provinces had low scores. Notably, Guizhou and Yunnan are the worst due to low economic development levels and harsh natural conditions. In 2014, we observe that a few provinces had improved well-being, notably middle provinces, such as Shanxi, Henan, Anhui, Hubei, and Jiangxi. It should be noted that Shaanxi significantly improved its well-being, which was mainly attributed to culture and education scores and relatively high social welfare. In 2017, the well-being of all provinces significantly improved, except Guizhou. On the one hand, from 2011 onwards, China accelerated its economic growth and meanwhile has attached great importance to well-being by increasing investment in social welfare, education, and environmental protection. On the other hand, Guizhou has long been experiencing a low economic growth rate. In addition, harsh natural conditions make it difficult to develop industrialization and increase local incomes, which is responsible for low well-being. In 2020, most provinces witnessed great improvements in well-being. Notably, the three northeastern provinces were prominent since they are high scores. Moreover, western provinces, such as Inner Mongolia, Ningxia, Gansu, and Qinghai, also experienced great improvements. However, Guizhou was still the lowest well-being province in China.

### 3.5. Moran’s I Test Results

**Figure 6:**
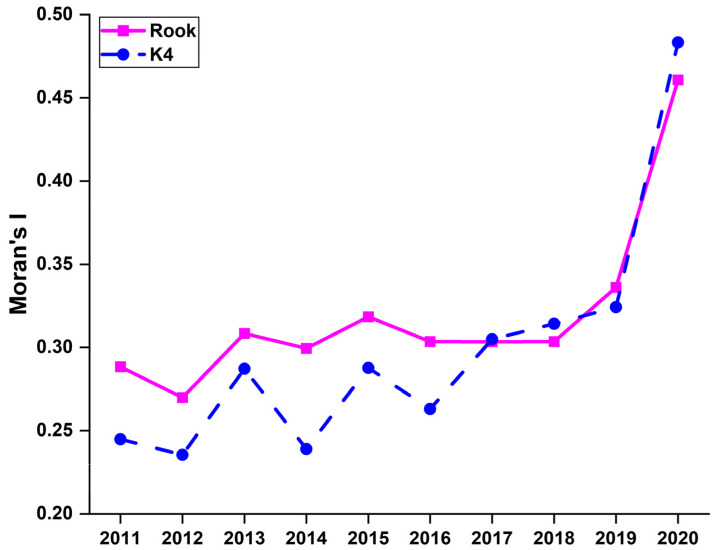
Moran’s *I* values with Rook and K4 matrices from 2011 to 2020.

Figure 5 shows that the well-being scores of 30 provinces are not evenly distributed in space and they present spatial clustering. Hence, we applied Moran’s *I* test to verify if there is spatial autocorrelation. The hypothesis of random spatial distribution can be strongly rejected, indicating significant spatial autocorrelation. In addition, the Moran’s *I* values from 2011 to 2020 with the Rook and K4 spatial weights matrices are plotted in Figure 6.

We find that from 2011 to 2019, overall Moran’s *I* values with the Rook matrix present a slightly increasing trend with fluctuations, and dramatically went up in 2020. The main reason is that the COVID−19 pandemic affected various indicators of the systems, which in turn strengthened spatial clustering. Specifically, all economic indicators have been deeply affected by the COVID−19 pandemic. However, economic performance may also exist with obvious spatial differences. The economically developed eastern region had a stronger ability to possibly eliminate the negative impact of the pandemic while the underdeveloped western region had to make great efforts to fight against the pandemic and was plagued with the negative consequences, which led to the economic slowdown. In this sense, the gap between the east and the west has widened and the spatial clustering of well-being has strengthened. Moreover, a similar conclusion can also be made when adopting the k nearest neighbor matrix. From the above analysis, it follows that the well-being scores of 30 provinces exhibit significant spatial spillovers, which should be incorporated when examining the convergence.

## 4. Results of Convergence Analysis

### 4.1. σ Convergence Analysis

**Figure 7:**
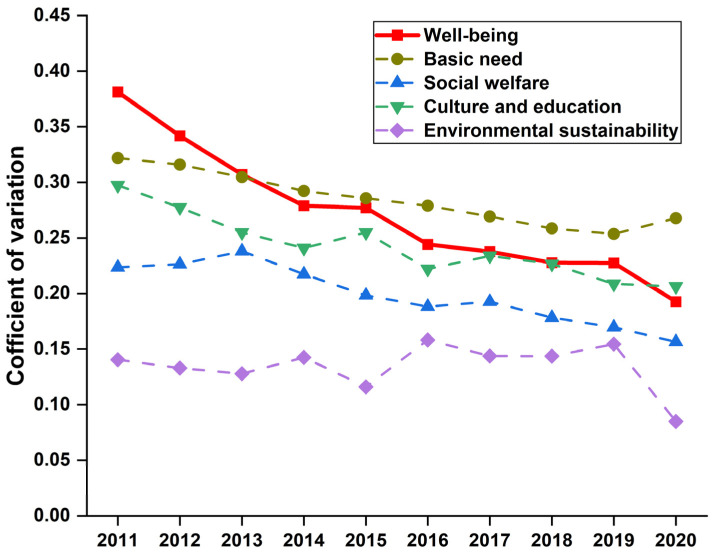
CV values of four subsystems and well-being from 2011 to 2020.

From the above discussions, it is safe to say that there is significant regional variability in the scores of four subsystems and well-being across the 30 provinces. The coefficient of variation (CV), defined as the ratio of standard deviation to mean, is used to measure the dispersion of well-being scores, which can be usually adopted to test if they can converge to a common steady state. Specifically, a larger CV value indicates a wider gap in the well-being scores and vice versa. The CV values of four subsystems and well-being across 30 Chinese provinces from 2011 to 2020 are plotted in Figure 7.

Overall, the CV values of the four subsystems presented a decreasing trend from 2011 to 2020 with fluctuations, indicating that the differences in the scores of the four well-being subsystems became narrow. However, we notice that the CV value of the basic needs subsystem in 2020 sharply went up. The main reason is that the COVID−19 pandemic widened the gaps in cross-province incomes. Notably, it caused numerous economic losses in the underdeveloped western provinces. Moreover, much attention is paid to the CV value of the environmental sustainability subsystem this fluctuated more often. An underlying reason is that emissions of industrial pollutants varied year by year. Notably, in 2016 pollutants substantially increased due to preferential policies at the beginning of the 13th five-year plan, which caused a sharp increase in the CV value. As environmental investment increased and pollutants declined in all provinces, the CV value continued to decrease from 2017 to 2020. Last, we find that the CV value of well-being also presented a decreasing trend over the sample period, indicating that the gap in cross-province well-being scores narrowed. In other words, there is *σ* convergence.

**Figure 8:**
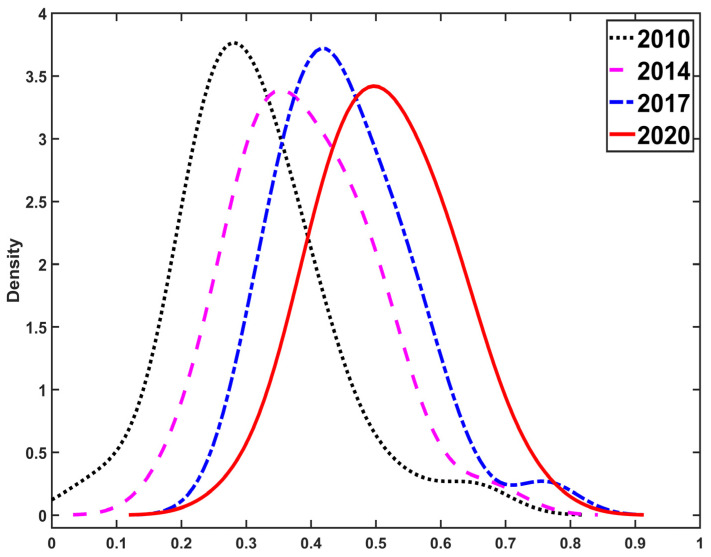
Kernel density estimations results of well-being scores in 2011, 2014, 2017, and 2020.

Then, kernel density estimations results are given in Figure 8 in order to better explore how the well-being scores of 30 provinces change over time.

From Figure 8, we observe that the curve moved from left to right from 2011 to 2020, indicating that overall national well-being improved over time. The left tail of the distribution became shortened and also moved from left to right, implying that provinces with low well-being scores had significantly improved over the decade. On the other hand, the right tail of the curves slightly extended, suggesting that provinces with high well-being scores had improved a little. Notably, we notice that the right tail in 2020 was shortened compared with that in 2017. The main reason is that the COVID−19 pandemic affected provinces with high well-being scores.

### 4.2. β Convergence Analysis

In this subsection, we analyze β convergence of cross-province well-being scores. The results of the traditional absolute and conditional β convergence models are presented in Table 2 and Table 3, respectively.

Models (1)–(5) are the results of the convergence of cross-provinces’ well-being and four subsystem scores. We first focus on Model (1). The estimated coefficient is found to be significant and negative (−0.126), indicating that the differences in the well-being scores of 30 provinces will converge to a common state. In other words, low well-being provinces will catch up with those with high well-being scores in the future. Similarly, we also observe that the coefficients of Models (2)–(5) are significantly negative, implying that the gaps among the four subsystems will become narrow. To summarize, from the above analysis, it follows that absolute β convergence can be confirmed.

Next, we pay attention to the results of the conditional β convergence model, which is shown in Table 3. We find that the five estimated coefficients are significant and negative, also indicating conditional β convergence. Differently, the magnitude of the coefficients of conditional β convergence is larger (absolute value) than those of absolute β convergence in Table 2. The main reason is that absolute β convergence assumes that well-being scores converge to the same growth path in all 30 provinces while in contrast, conditional β convergence refers to convergence conditional on province-specific characteristics, not the initial level of well-being. Hence, the convergence speed of conditional β convergence is fast than that of absolute β convergence.

Last, we incorporated spatial spillovers to build spatial conditional β convergence. The results are summarized in Table 4.

We find that the spatial autoregressive coefficient *ρ*, is significant and positive, indicating spatial spillovers, consistent with Moran’s *I* results in Figure 6. Moreover, the β coefficient in the spatial conditional β convergence model is also significantly negative, indicating that conditional β convergence has been confirmed. Similarly, it can also be found in the four subsystems, namely, Models (12)–(15). However, the coefficient *ρ*, in Models (12) and (13) is highly insignificant, though positive, indicating that the scores of the basic needs and social welfare subsystems are randomly distributed in space. To summarize, from Table 2, Table 3 and Table 4, both absolute and conditional β convergence have been verified.

## 5. Conclusions

In this research, we built a comprehensive evaluation system including four subsystems and 36 indicators and adopted the entropy weight method to calculate the well-being scores of 30 provinces in China from 2011 to 2020. Then, the spatiotemporal distribution and variations of the well-being scores of 30 provinces were analyzed and discussed using various statistical methods. Finally, the coefficient of variation, kernel density estimation, and spatial econometric models were employed to test if there is *σ* and β convergence in the cross-province well-being scores. The main findings of this study are as follows. (1) The average scores of the well-being and the four subsystems presented an increasing trend from 2011 to 2020, indicating that national well-being has improved significantly in the recent decade. However, the scores of the basic needs subsystem in 2020 sharply went down due to the impact of the COVID-19 pandemic on the economy. (2) Most provinces had both advantages and disadvantages in terms of the four subsystems. High well-being provinces, mainly located in the coastal eastern region (e.g., Beijing, Tianjin, Liaoning, Jiangsu, Fujian, Shanghai, Zhejiang, and Shandong), had fewer disadvantages. Yunnan and Guizhou, the worst well-being provinces, had the lowest scores, which left them at the bottom of the ranking. (3) The well-being scores of 30 provinces were not randomly distributed in geography, indicating spatial spillovers, which was confirmed by the results of Moran’s *I* test. In addition, the results showed that the spatial clustering is strengthened over time. (4) The CV values implied that the gap in the well-being across provinces became narrowed from 2011 to 2020, which was consistent with the kernel density estimation results. It implied that there was *σ* convergence. In addition, low well-being provinces had significantly improved in the recent decade while high well-being provinces in 2020 were more affected by the COVID−19 pandemic. On the other hand, absolute and conditional β convergence had been verified through traditional and spatial convergence models.

Policy implications based on the main findings can be proposed. Since most provinces have strengths and weaknesses, the improvements of disadvantages should be given the first priority notably in the context of the specific characteristics of these provinces. The low well-being provinces were mainly located in the large area of western China. Harsh natural conditions make them difficult to improve well-being levels on their own, for example, Yunnan and Guizhou provinces. In other words, they have long been dependent on financial fund allocation from the central government. Targeted and precise poverty alleviation and improvement of living standards can greatly benefit the well-being levels of these western provinces. The economically developed eastern provinces should maintain and strengthen their advantages, but pay much more attention to disadvantages, notably food supply. We observed that several eastern provinces had a shortage of per capita food products. Hence, targeted policies to improve the disadvantages will enable a balanced development of well-being. On the other hand, several western provinces had advantages in culture and education, for example, Shaanxi had advantages because of numerous museums and universities, but had disadvantages in basic needs. Hence, the Shaanxi government should focus on improving per capita food products. Moreover, Ningxia outperformed the other western provinces and had substantially improved well-being, which is mainly attributed to the increased investment in social welfare and culture and education in recent years. In addition, it should also pay due attention to the basic needs and environmental sustainability indicators so that it can develop in a balanced way. The convergence results imply that the low well-being provinces will catch up with those with high well-being scores. However, at present, there are obvious spatial disparities in well-being scores among Chinese provinces. Hence, the Chinese central and local governments should implement accurate policies and measures to accelerate the realization of narrowing the cross-province well-being gap in the future.

## Figures and Tables

**Table 1:** Indicators for evaluating human well-being.

Subsystem	Indicator	Unit	Positive/ Negative
Basic needs	Per capita gross provincial product	Yuan	Positive
	Per capita consumption expenditure of households	Yuan	Positive
	Per capita water resource	m^3^	Positive
	Per capita output of grain	kg	Positive
	Per capita output of poultry	kg	Positive
	Per capita output of meat	kg	Positive
	Per capita aquatic products	kg	Positive
Social welfare	Number of deaths in traffic accidents per 10 thousand	capita	Negative
	Number of participants in work-related injury insurance per 10 thousand	capita	Positive
	Number of beneficiaries in work-related injury insurance per 10 thousand	capita	Positive
	Number of beneficiaries in birth insurance per 10 thousand	capita	Positive
	Ratio of participants of basic endowment insurance for worker	%	Positive
	Number of beds of health care institutions per 10 thousand	bed	Positive
	Number of health technical personnel per 10 thousand	capita	Positive
	Ratio of inpatients per 10 thousand	%	Positive
	Divorce rate	%	Negative
	Ratio of urban residents entitled to minimum living allowance	%	Positive
	Ratio of rural residents entitled to minimum living allowance	%	Positive
	Unemployment rate	%	Negative
Culture and education	Number of school enrolment population by higher education per 10 thousand	capita	Positive
	Number of school enrolment population by senior secondary education per 10 thousand	capita	Positive
	Number of higher education institutions	institution	Positive
	Ratio of the number of students enrolled in higher education institutions	unit	Positive
	Number of museums per million	unit	Positive
	Number of public libraries per million	unit	Positive
Environmental sustainability	Per capita investment in the treatment of industrial pollution	Yuan	Positive
	Per capita investment in the treatment of wastewater	Yuan	Positive
	Per capita investment completed in the treatment of waste gas	Yuan	Positive
	Per capita investment completed in the treatment of other pollution	Yuan	Positive
	Per capita SO_2_ emissions	kg	Negative
	Per capita NO_x_ emissions	kg	Negative
	Per capita area of afforestation	m^2^	Positive
	Per capita manual planting	m^2^	Positive
	Per capita wastes collected and transported	kg	Positive
	Volume of harmless disposal of wastes	ton	Positive
	Harmless disposal rate of municipal waste	%	Positive

**Table 2:** Results of absolute β convergence models.

Model	Model (1)	Model (2)	Model (3)	Model (4)	Model (5)
Variables	WB	BN	SW	CE	ES
L. LogWB	−0.126 *** (0.013)				
L. LogBN		−0.048 *** (0.007)			
L. LogSS			−0.042 *** (0.011)		
L. LogCE				−0.051 *** (0.014)	
L. LogES					−0.246 *** (0.031)
Constant	−0.058 ***	−0.029 ***	−0.010	−0.028 *	−0.232 ***
	(0.013)	(0.009)	(0.010)	(0.016)	(0.032)
Obs.	270	270	270	270	270
R^2^	0.260	0.168	0.049	0.050	0.195

Note: Standard errors are in parentheses. *** *p* < 0.01, * *p* < 0.1.

**Table 3:** Results of conditional β convergence models.

Model	Model (6)	Model (7)	Model (8)	Model (9)	Model (10)
Variables	WB	BN	SW	CE	ES
L. LogWB	−0.411 *** (0.038)				
L. LogBN		−0.142 *** (0.045)			
L. LogSW			−0.180 *** (0.044)		
L. LogCE				−0.266 *** (0.042)	
L. LogES					−0.635 *** (0.055)
Constant	−0.400 ***	−0.154 **	−0.141 ***	−0.288 ***	−0.723 ***
	(0.048)	(0.072)	(0.042)	(0.053)	(0.067)
Obs.	270	270	270	270	270
R^2^	0.435	0.274	0.171	0.339	0.439

Note: Standard errors are in parentheses. *** *p* < 0.01, ** *p* < 0.05.

**Table 4:** Results of spatial conditional β convergence models.

Model	Model (11)	Model (12)	Model (13)	Model (14)	Model (15)
Variables	WB	BN	SW	CE	ES
L. LogWB	−0.407 *** (0.037)				
L. LogBN		−0.138 *** (0.044)			
L. LogSW			−0.181 *** (0.044)		
L. LogCE				−0.256 *** (0.041)	
L. LogES					−0.632 *** (0.054)
*ρ*	0.125 *** (0.086)	0.065 (0.092)	0.021 (0.094)	0.173 *** (0.085)	0.096 (0.088)
Obs.	270	270	270	270	270
Pseudo R^2^	0.259	0.189	0.1225	0.168	0.209

Note: Standard errors are in parentheses. *** *p* < 0.01.

## Data Availability

The data presented in this research are available on request from the corresponding author. All data in our study come from “China Statistical Yearbooks”, which can be obtained from http://www.stats.gov.cn/tjsj/ndsj/.

## Statement

This article is in memory of the second author, Chen Yuan, who passed away on 30 December 2022 at the age of 25. He is a very good person, friend, and student of ours.
